# “This is Just a Prototype”: How Ethics Are Ignored in Software Startup-Like Environments

**DOI:** 10.1007/978-3-030-49392-9_13

**Published:** 2020-05-06

**Authors:** Ville Vakkuri, Kai-Kristian Kemell, Marianna Jantunen, Pekka Abrahamsson

**Affiliations:** 6 University of Oslo, Oslo, Norway; 7 Monash University, Clayton, VIC Australia; 8 IT University of Copenhagen, Copenhagen, Denmark; 9 University of British Columbia, Vancouver, BC Canada; University of Jyväskylä, PO Box 35, 40014 Jyväskylä, Finland

**Keywords:** Artificial intelligence, AI ethics, AI development, Practices, Responsibility, Accountability, Transparency, Case study

## Abstract

Artificial Intelligence (AI) solutions are becoming increasingly common in software development endeavors, and consequently exert a growing societal influence as well. Due to their unique nature, AI based systems influence a wide range of stakeholders with or without their consent, and thus the development of these systems necessitates a higher degree of ethical consideration than is currently carried out in most cases. Various practical examples of AI failures have also highlighted this need. However, there is only limited research on methods and tools for implementing AI ethics in software development, and we currently have little knowledge of the state of practice. In this study, we explore the state of the art in startup-like environments where majority of the AI software today gets developed. Based on a multiple case study, we discuss the current state of practice and highlight issues. The cases underline the complete ignorance of ethical consideration in AI endeavors. We also outline existing good practices that can already support the implementation of AI ethics, such as documentation and error handling.

## Introduction

AI systems have become increasingly common in software engineering projects [1]. While much of the media attention is on flashier systems such as autonomous vehicles, less high-profile AI systems such as decision-making support systems have become increasingly widespread in various organizations. AI systems often operate under the surface in the form of e.g. recommendation algorithms, making the high-profile systems in the middle of the media hype only the tip of the iceberg.

Over the last two decades, progress on AI has been accelerating rapidly. AI systems are now widely used in various areas and for various purposes. Examples include medical systems [2], law enforcement [3], and manufacturing industries and industry 4.0 [4], among numerous others. As the field progresses, the already impressive potential of AI systems becomes even larger, including applications such as general AI systems, the likes of which are already being developed by the technology giants such as Alphabet. It is exactly because of this impressive potential and impact of these systems, especially in the future, that their potential negative impacts should also discussed more.

AI systems are ultimately still software. They are affected by largely the same requirements as any other software system. AI development projects are still for the most part conventional software engineering, with machine learning related tasks only comprising a small portion of these projects [5].

However, AI systems are unique in terms of their effects on various stakeholders to the point where they can even exert society-wide influence. Moreover, these stakeholders often have little power in opting out of using these systems. E.g. it is difficult to avoid having a firm filter your job application using AI or trying to avoid being monitored using AI-based surveillance systems if such systems are in place in the area.

Various system failures have already highlighted some of the potential issues these systems can have in practice. Past incidents that have received global media coverage, even smaller incidents can be costly for the affected organization(s). For example, the national Finnish broadcasting company, Yle^1^, utilized AI for moderation purposes in its services. Having already changed its processes to suit the automation of the moderation, the organization ultimately ran into problems with the AI moderator system. Though the software was working fine on the technical level, the socio-ethical issues forced the organization to revert back to human moderators.

Many of these issues are ultimately rooted in ethics. AI ethics has thus become a new non-functional requirement to address; an -ility among the likes of quality, maintainability, and scalability. Existing methods have focused on tackling these functional and non-functional requirements. However, no such methods currently exist for AI ethics [6], with the existing tools and methods largely being technical and limited to narrow contexts in ML as opposed to being project-level methods.

In the absence of methods, how are ethics currently implemented? Much of the current literature in the area has been theoretical, and our understanding of the state of practice in AI ethics is currently lacking. [6] AI ethics literature discusses various aspects of AI ethics that should be taken into account, but bridging the gap between research and practice in the area remains an on-going challenge [7, 8]. Guidelines for implementing AI ethics exist, but their effect on the start of practice remains unknown.

Thus, to begin bridging this gap in the area, we conduct an empirical study to help us understand the current state of practice. We do so by means of a multiple case study of three projects focusing on healthcare systems. The goal of this study is two-fold: (1) to help us understand the current state of practice in AI ethics; and (2) to discover existing good practices that might help in implementing AI ethics. Out of these two goals, the first is a theoretical contribution while the second one is a practical one. The specific research question of the paper is as follows:

**RQ:** how are AI ethics taken into consideration in software engineering projects when they are not formally considered?

## Related Work: The Current State of AI Ethics

Ethics in software development and interactive systems design in general has a history of over 30 years. For example, Bynum [9] introduced the idea of adapting human values in design before the rise of human computer interaction and other human-centric paradigms. Theoretically grounded approaches such as Value Sensitive Design (VSD) and its variants have provided tools to design technology that takes into account human values in the design process [10, 11].

As more progress is made in the field of AI systems, old theoretical scenarios in AI ethics are slowly becoming reality. This calls for new methods to manage the ethical issues arising from these new systems [7, 12]. Indeed, Vallach and Allen [12] argue that AI and AI-based systems produce new requirements to consider. Specifically, they propose that designers implicitly embed values in the technologies they create [12]. AI and other complex systems force designers to consider what kind of values are embedded in the technologies and also how the practical implementation of these values could be carried out and how these systems could be governed [13].

Yet, little is currently known about software development practices and methods in the context of AI ethics, as empirical studies in the area are scarce. Our results from an existing study suggest that AI ethics are seldom formally implemented in SE projects, [14]. Similarly, there are currently no project-level methods that could aid in implementing AI ethics [6]. On the other hand, various tools that can support specific elements of AI ethics do exist, such as tools for managing machine learning [6]. However, they do not help developers implement AI ethics in general.

In this light, it can be said that AI ethics has hardly been incorporated into mainstream SE literature yet. The reason why AI Ethics has received little attention in the prior engineering literature is three-fold: 1) Prior research has been predominantly philosophical, 2) the field has not sensed the need to address ethical concerns and 3) thus it has not been part of the education system.

Though some practice-focused research does exist (e.g. [15]), most of the research on AI ethics has been conceptual and theoretical in nature. These studies have e.g. focused on defining AI ethics in a practical manner through various constructs in the form of values. For the time being, this discussion on defining AI ethics has come to center around four values: transparency [16, 17], accountability [8, 16], responsibility [16], and fairness (e.g. [18]). Not all four of these values are universally agreed to form the core of AI ethics, however, as we discuss in the following section while presenting our research framework.

Following various real-life incidents out on the field (e.g. Amazon’s biased recruitment AI^2^), AI ethics has also begun to spawn public discussion. This has led to governments, standardization institutions, and practitioner organizations reacting by producing their own demands and guidelines for involving ethics into AI development, with many standards and regulations in the works. Countries such as France [19] and Germany [20] have emphasized the role of ethics in AI, and on an international level the EU began to draft its own AI ethics guidelines which were presented in April 2019 [21]. Moreover, ISO has founded its own ethical, trustworthy AI in ISO/IEC JTC 1/SC 42 Artificial intelligence subcommittee [22]. Finally, some larger practitioner organizations have also presented their own guidelines concerning ethics in AI (e.g. Google [23] and Microsoft [24] guidelines).

Thus far, these various attempts to bring this on-going academic discussion out on the field have been primarily made in the form of guidelines and principles. Out of these guidelines, perhaps the most prominent ones up until now have been the IEEE guidelines for Ethically Aligned Design (EAD), born from the IEEE Global Initiative on Ethics of Autonomous and Intelligent Systems alongside its IEEE P7000™ Standards Working Groups, which were branded under the concept of EAD [8].

Existing literature has shown us that guidelines and principles in the field of ICT ethics do not seem to be effective. Mittelstadt [25] argue that AI developers lack the professional norms and methods to translate principles into practice in successful way. To this end, McNamara et al. [26] also argue based on empirical data that the ACM ethical guidelines^3^ had ultimately had very little impact on developers, who had not changed their ways of working at all. In this light, this is likely to be the case with the aforementioned AI ethics guidelines as well, as Mittelstadt suggest [25]. This notion is further supported by Morley et al. [6] who argue that developers focused on practicality are unlikely to adopt them when the competitive advantage of EAD is unclear.

## Research Model

**Fig. 1.**
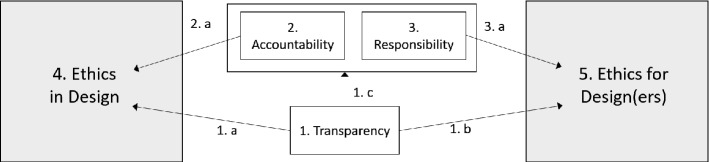
Research framework

To assist in the data collection and analysis in this study, we devised a research framework based on prominent literature in the area. This research framework and the justifications behind it are further discussed in an existing paper [27] (Fig. 1).

As the basis of the framework, we utilized the ART principles of Dignum [16], which consist of Accountability, Responsibility, and Transparency. These have been central constructs in the area, having also been featured in the EAD guidelines of IEEE.

*Transparency* is required for accountability and responsibility (line 1.c), as we must understand why the system acts in a certain fashion, as well as who made what decisions during development in order to establish accountability [17]. Whereas accountability can be considered to be externally motivated, closely related but separate construct responsibility is internally motivated. The concept of accountability holds a key role in aiming to prevent misuse of AI and in supporting wellbeing through AI [8].

*Accountability* refers to determining who is accountable or liable for the decisions made by the AI. Dignum [16] in their recent works defines accountability to be the explanation and justification of one’s decisions and one’s actions to the relevant stakeholders. In the context of this research framework, accountability is used not only in the context of systems, but also in a more general sense. We consider, e.g., how various accountability issues (legal, social) were considered during development.

Dignum [16] defines *responsibility* as a chain of responsibility that links the actions of the systems to all the decisions made by the stakeholders. We consider it to be the least accurately defined part of the ART model, and thus have taken a more comprehensive approach to it in our research framework. According to the EAD guidelines, responsibility can be considered to be an attitude or a moral obligation for acting responsibly [8] A simplified way of approaching responsibility would be for a developer to ask oneself e.g. “would I be fine with using my own system?”.

In addition to the ART principles, we utilized the three AI ethics categories presented by Dignum [28] to make these constructs more practical. Dignum suggests that AI ethics can be divided into:

- Ethics by Design (integration of ethical reasoning capabilities as a part of the behaviour of artificial autonomous system, e.g. ethical robots);
- Ethics in Design (the regulatory and engineering methods supporting ethical implications of AI systems); and
- Ethics for Design: (codes of conduct, standards, and certification processes that ensure the integrity of developers and users) [28].

In this paper, we focus on the ethically aligned *development process*, and therefore the last two categories were included into the research framework.

Finally, aspects of commitment were utilized in the framework to aid data analysis. Specifically, we utilized the commitment net model of Abrahamsson [29] to approach the implementation of ethics into practice and have an explaining theoretical framework to examine ethics role to developers. From this model, we focused on concerns and actions. Concerns were analyzed to understand what ethical issues were of interest to the developers. Actions were then studied to understand how these concerns were actually tackled, or whether they were tackled at all.

In commitment net model, actions are connected to concerns because when actions are taken, they are always driven from concerns [29]. On the other hand, however, concerns can exist without any actions taken to address them. The dynamic between actions and concerns was considered a tangible way to approach the focus of this study: practices for implementing AI ethics. Developers actions could be likened to practices that were taking during the development. On the other hand, analyzing the concerns that developers have opens a view to understanding e.g. whether the developers perhaps wanted to implement ethics but were unable to do so.

## Study Design

This section is split into three subsections. First, we discuss the cases of the case study. In the second and third ones we discuss the data collection and analysis, respectively.

### Cases

We conducted a multiple case study featuring three case projects. In all of the case projects, AI systems were being developed for the healthcare sector. These cases are outlined in the table below (Table 1). We chose to utilize a qualitative case study approach due to the exploratory nature of the topic, as the research area is novel as far as empirical studies are concerned.

**Table 1.** Descriptions of each case

Case	Example	Font size and style
A	Statistical tool for detecting social marginalization	Data Analyst [R1], Consultant [R2], Project Coordinator [R3]
B	Speech recognition and NLP based tool for diagnostics	Developer [R4], Developer [R5], Project Manager [R6]
C	NLP based tool for indoor navigation	Developer [R7], Developer [R8]

Healthcare cases were selected due to the assumption that ethical consideration would be more common in healthcare-related projects due to the nature of the area in closely dealing with human well-being (e.g. the tradition of bio and medical ethics). Indeed, healthcare systems can, for example, influence the decisions made by doctors or their patients related to the health of the patients. Moreover, due to the emphasis on tax-funded public healthcare in Finland, where the cases were from, the area is particularly regulated. These regulations impose some ethical requirements on software systems as well, especially in relation to handling patient data, which is considered particularly sensitive data from a legal point of view.

In the paper title, we characterize these case projects as being startup-like because the projects shared various characteristics typically associated with software startups. First, agile methods were commonly utilized in the projects. Secondly, the projects were all characterized by notable time pressure. Thirdly, the projects operated with scarce resources. Fourthly, the cases were centered around the development of functional prototypes, which were intended to as proof-of-concept type artifacts. However, the prototypes were being developed with real customers and tested in practice. Finally, the projects exhibited exploratory approaches that focused on experimentation.

Currently, much of the on-going AI development is happening in startups [1], even if the multinational organizations receive much media coverage in relation to AI. In characterizing them as startup-like, we consider them to be representative of the current AI development projects.

### Data Collection

Data from the cases were collected using semi-structured interviews [30]. This interview strategy enabled the interviews to be conducted in a way that allowed for flexibility from the interview questions, but without steering too far from the topic. The interview instrument used in the interviews can be found externally as a reference^4^. All interviews were conducted as F2F interviews and the audio was recorded for transcription. The analysis was conducted using the transcripts. The interviews were conducted in Finnish. This was done so that the respondents would not give shorter responses due to being uncomfortable with communicating in English, especially while being on record.

The respondents from the cases were either developers or managers. As we wanted to focus on development practices and project issues, we focused on the personnel directly involved with the practical development issues in the projects. The respondents are outlined in the table in the previous subsection. In terms of experience, respondents 4, 5, 7, and 8 were junior developers. Respondents 3 and 6, on the other hand, were senior developers. Respondent 1 was a junior data scientist.

### Data Analysis

We analyzed the data in two phases. First, we utilized a grounded theory (Heath [31]) inspired approach to code the transcripts quote by quote for each interview. This process was carried out iteratively as the list of codes was updated during the process. This approach was chosen due to the lack of existing studies on the current state of practice in the area.

In the second phase, we utilized the commitment net model of Abrahamsson [29] to then further analyze and categorize the coded content. We utilized the model by focusing on the concerns and actions of the developers. The concerns and actions of each respondent were compared across cases in search of recurring concerns and actions between cases and respondents. By evaluating the relationships between the actions taken in development the development process and the concerns of the developers, we could better understand the motivation behind the actions. Similarly, we could also see which concerns did not lead to any actions, pointing to a lack of commitment towards tackling those concerns.

The data were then compared with the research framework again to evaluate how AI ethics were implemented in each project. Actions were the emphasis here, as the focus of this study was on tangible implementation of AI ethics and how it was carried out in terms of tools, practices, or methods. However, we also highlighted interesting findings in relation to the mere concerns related to AI ethics.

## Empirical Results

This section is split into four subsections. The first three feature the analysis split between the accountability, responsibility and transparency constructs. The final subsection summarizes the analysis. We highlight our findings as Primary Empirical Conclusions (PECs). During the analysis, we use quotes from the interviews to elaborate on the topic at hand. However, it should be noted that the conclusions are not drawn merely based on these individual citations.

### Responsibility

The concerns of the developers related to responsibility were varied, but ultimately detached from practice as far as concerns related to AI ethics were considered. The concerns the developers discussed in relation to responsibility were simply very practical concerns related to internal project matters or delivering a high quality product:

“Responsibility on reporting and keeping the project on schedule” (R6)

**PEC1.** Developers feel most responsibility towards tackling problems related to software development, such as finding bugs, meeting project goals.

On the other hand, as the interviews progressed, the developers did also express some concerns towards various ethical issues. However, these concerns were detached from their current work. They did not affect the way they worked, and the developers felt that these types of concerns were not relevant during development. The presence of concerns in the absence of actions to address those concerns pointed towards a lack of commitment on this front.

“It is just a prototype” (R8)

“I do my best” (R5)

“But this is a prototype, an experiment, just to show people that you can do this type of thing. This doesn’t really have any responsibility issues in it.” (R1)

**PEC2.** On a personal level, developers are concerned about the ethical aspects of product development. However, little is done to tackle these concerns.

Furthermore, it was evident that in none of the cases had the hypothetical effects of the system on the stakeholders been discussed. To give a practical example, a system potentially affecting memory illness diagnoses clearly has various effects on its potential users, especially when the test can be taken without supervision. Yet, the developers of this particular tool also felt that their users would not be curious about the workings of the system. They considered it sufficient if the responsibility was outsourced to the user and it was underlined that the system does not make the diagnosis but simply advises doctors.

The developers did not consider the potential harm of the system past the tangible, physical harm potential of the systems. For example, stress or other negative effects on users and other stakeholders were not considered. In all three cases, the respondents did not consider the system to have had any potential of causing physical harm, and thus did not consider the system to have any notable harm potential at all.

“Nobody wants to listen to ethics-related technical stuff. No five hour lectures about it. It’s not relevant to the users” (R5)

“I don’t really understand what it [responsibility] has to do with product development. We developers are all responsible.” (R7)

“What could it affect… the distribution of funds in a region, or it could result in a school taking useless action… it does have its own risks, but no one is going to die because of it” (R1)

**PEC3.** Responsibility of developers is unclear.

### Transparency

Case A highlighted the potential importance of mathematical expertise. The team had internal mathematical capabilities that allowed them to develop their own algorithms, as well as to better understand third party components, in order to have achieve a higher standard of transparency. They utilized algorithms they were familiar with and which they understood on an in-depth level. Thus, the team considered themselves to be able to understand why the system made certain decisions in certain situations. This underlines the importance of mathematical skills in preventing the birth of black boxes in AI development.

“In that sense it’s not really a black box as we can understand what’s going on in there just fine, and we can show the nodes and what affects them. It’s a very transparent algorithm.” (R3)

The other two cases utilized existing AI solutions. They did not have an in-depth understanding of the technologies they were utilizing, which resulted in their systems being (partially) black boxes. They understood any components created by the team but did not have a full understanding of the third party components they had used as a base. This presents problems for feature traceability.

**PEC4.** Black box systems are a typical issue in AI development.

Even though transparency of algorithms and data was not present in two of the cases, the developers in case B nonetheless acknowledged its potential importance However, as it was not considered a formal requirement in the projects, the managers did not devote resources towards pursuing it. Even in case A, transparency was not produced as a result of ethical goals but out of business reasons.

“We have talked about the risks of decision-making support systems but it doesn’t really affect what we do” (R5)

**PEC5.** Developers recognize transparency as a goal, but it is not formally pursued.

On the other hand, in relation to transparency of systems development, all three cases displayed transparency. By having formal decision-making strategies, they were able to keep track of higher-level decisions related to the system. Through proper documentation, they were able to keep track of decisions made on the code level. Version control also assisted in this regard, making it clear who made what changes and when in retrospect. There were thus various existing practices that produced transparency of systems development. Two of the cases also acknowledged the effects of team size on transparency of systems development. They noted that, in addition to documentation practices, the small team size itself made it easy to keep track of the actions of individual developers even in an ad hoc manner.

**PEC6.** Established SE practices, such as code documentation and code review, support transparency of systems development.

### Accountability

Some aspects of accountability were clear points of focus in the projects, namely ones related to security in terms of general information security as well as data management. The respondents were aware of being in possession of personal data, given that they developed healthcare solutions, and were concerned with keeping it secure. They mentioned taking measures to keep the data secure from potentially malicious actors, and they were aware that they would have to take measures to act in accordance with laws and regulations in the area. However, in some cases they had not done so yet.

“It’s really important how you handle any kind of data, that you preserve it correctly, among researchers, and don’t hand it out to any government actors. For example, many of the data packages have kind of interesting data and it can’t get into the wrong hands. I personally can’t see any way to harm anyone with the data we have though” (R2).

“We haven’t really paid much attention to the [data] safety aspects yet… it hasn’t really been a main focus. There’s probably a lot of things we have to take into account [eventually]” (R5).

The ethical concerns they had in relation to accountability were in general largely related to existing areas of focus in software development. For example, error handling was one aspect of accountability the respondents were particularly concerned with. This was tied with their goal of making quality software, which they considered their responsibility as professionals. The respondents could, to this end, discuss what tangible practices they utilized to deal with error handling.

**PEC7.** Developers feel accountable for error handling and have the means to deal with it.

However, error handling was largely considered from the point of view of writing code and testing it in a laboratory setting. I.e. the system was considered error free if there were no red lines in the code in the IDE during development. Only case company B discussed measures they had taken to monitor errors in use. Furthermore, potential misuse (e.g. a prankster drawing a horizontal white line on the pavement to intentionally confuse autonomous vehicles) and error scenarios during the operational life of the system had not been actively considered in any of the case projects.

“The calculations are made in the algorithms, so it doesn’t really make mistakes” (R2)

**PEC8.** Product misuse and error scenarios are only considered during development. They are not considered in terms of the future operational life of the system out on the field.

Due to the nature of machine learning, AI systems learn as they are taught with new data or as they collect it themselves while operating out on the field. From this arises the potential issue of unexpected behavior as a result of machine learning. None of the respondents had made plans to tackle potential unexpected behavior during the operational life of their system, should such behavior arise. In only one of the projects was the possibility directly acknowledged:

“We just put it up for end-users to test and note that this is still being developed” (R7).

**PEC9.** Developers do not have plans to deal with unexpected behavior of the system resulting from e.g. machine learning or the future expansion of the use context of the system.

### Summary of Findings

Past the ART constructs, we highlight some commonalities between the cases on a more general level while summarizing our findings. In none of the cases were ethics implemented by following a formal method or tool, nor were ethical issues considered directly as ethical issues. Rather, any ethical issues tackled in the projects were tackled for practical reasons (e.g. error free software is beneficial from the point of view of customer relations). Nonetheless, some of the ethical issues such as error handling and transparency of systems development were tackled in a systematic manner through existing software engineering practices such as code documentation and version control.

On the other hand, though ethics were not taken into consideration on a project level, the respondents still exhibited some concern towards the potential socio-ethical issues in the systems. When prompted, they were able to come up with various negative effects the systems could have on different stakeholders. They considered these to be potential real issues, but did not have a way to address these concerns in the absence of tools, practices, and methods for doing so. Moreover, they seemed to realize these potential issues only after being directly asked about them in the interviews. This also points to a lack of tools to aid in ethical analyses.

## Discussion

In this section, we have collected all the Primary Empirical Conclusions (PEC) outlined in preceding analysis section into Table 2. We relate each of these findings to existing literature and discuss their implications in this section. We classify each of these PECs based on their contribution into either novel findings, findings that (empirically) validated existing literature, or findings that contradict existing literature.

**Table 2.** List of Primary Empirical Conclusions (PECs)

#	Theoretical component	Description	Contribution
1	Responsibility	Developers feel most responsibility towards tackling problems related to software development, such as finding bugs, meeting project goals	Empirical validation
2	Responsibility	On a personal level, developers are concerned about the ethical aspects of product development. However, little is done to tackle these concerns	Novel
3	Responsibility	Responsibility of developers is unclear	Novel
4	Transparency	Black box systems are a typical issue in AI development	Empirical validation
5	Transparency	Developers recognize transparency as a goal, but it is not formally pursued	Contradicts existing literature
6	Transparency	Established SE practices, such as code documentation and code review, support transparency of systems development	Empirical validation
7	Accountability	Developers feel accountable for error handling and have the means to deal with it	Empirical validation
8	Accountability	Product misuse and error scenarios are only considered during development. They are not considered in terms of the future operational life of the system out on the field	Contradicts existing literature
9	Accountability	Developers do not have plans to deal with unexpected behavior of the system resulting from e.g. machine learning or the future expansion of the use context of the system	Contradicts existing literature

Many of our findings underline a gap between research and practice in the area. Whereas research on AI ethics alongside various guidelines devised by researchers [8] and practitioners [23, 24] alike has discussed various ethical goals for AI systems, these goals have not been widely adopted out on the field. In this sense, we consider some of our findings (PECs 4, 5, 8, and 9) to contradict existing literature.

For example, extant literature has highlighted the importance of transparency of algorithms and data [15–17]. Without understanding how the system works, it is impossible to establish why it malfunctioned in a certain situation, which may e.g. be pivotal in understanding the causes of an accident that resulted in material damage [15]. Our findings point towards transparency being largely ignored as a goal (PEC5). Existing system components are utilized as black boxes, and developers do not see this as a notable problem (PEC4). We consider PEC5 to contradict existing literature in that existing literature has, on multiple occasions, highlighted the importance of transparency in AI systems. Yet, out on the field, this importance does not seem to be recognized to the point where it would result in changing development practices.

The situation is similar for tackling potential misuse of the systems, error handling during system operations, and handling unexpected system behavior (PEC8-9). These goals are included into the IEEE EAD guidelines [8]. However, none of the case companies took any measures to address these potential issues.

On a further note of transparency, however, the lack of emphasis placed on it is also curious in relation to feature traceability in SE. For decades, understanding the inner workings of the system was considered key in any SE endeavor. Yet, in the context of AI systems, the long-standing goal of feature traceability seems to be waning. Our findings point towards this being at least partially a result of a lack of mathematical understanding, as the one case company that considered their system to be fully transparent also noted that they fully understood the mathematics behind the algorithms they utilized. In using existing components in their systems, developers may not always understand the algorithms in these components. Indeed, in this vein, [32] noted that simply seeing the code is not enough if the algorithm is not understood, or the system is not understood as a whole.

Though we discovered various examples of ethics not being implemented, we also discovered that various existing and established SE practices can be used to implement AI ethics. Documentation, version control, and project management practices such as meeting transcripts produce transparency of systems development by tracking actions and decision-making (PEC6). Similarly, software quality practices help in error handling also in the context of AI ethics (PEC7), although they do not specifically account for the errors autonomous systems may face while operating out on the field. While discussing responsibility with the respondents, we also discovered that most of their responsibility was related to producing quality software and meeting project requirements. This validates existing literature in the area of SPI (e.g. Unterkalmsteiner, [33]).

Notably, we also discovered that the developers had ethical concerns towards their systems, which is a novel finding in this context (PEC2). Little is currently known about the state of practice out on the field, although a recent version of the EAD guidelines speculated about a gap in the area, which our findings support in relation to most aspects of AI ethics. Despite AI ethics largely not being implemented, our findings point towards it partially being a result of a lack of formal methods and tools to implement it.

In our data, the reason given by multiple respondents for not actively considering ethical issues was that they were developing a prototype. However, prototypes do influence the final product or service developed based by them, as shown by existing studies [34]. AI ethical issues should be tackled during earlier stages of development as well, seeing as many of them are higher-level design decisions (such as how to carry out machine learning in the system [15]), which can be difficult to undo later.

Following this study, as well as a past case study [14], we suggest that future research seek to tackle the lack of methods and tooling in the area. Though developers may be concerned about ethical issues, they lack the means to address these concerns. On the other hand, methods can also raise the awareness of developers in relation to AI ethics, creating concerns where there now are none. In creating these methods, we suggest exploring existing practices that can be used as is or tailored to implement AI ethics, as we have discussed here.

Given the amount of activity in AI ethics currently, with many governmental actors drafting their own AI ethics guidelines, likely followed by regulations, methods and tools will likely have practical demand in the future. Thus, even if one barrier to implementing AI ethics is currently the fact that it is seldom considered a requirement on a project level, regulations and laws can force organizations to take ethics into account. This would inevitably result in a demand for methods in this area, as well as the birth of various in-house ones.

Finally, in terms of limitations, the most notable limitations of the study stem from the data and the research approach. The qualitative multiple case study approach always poses problems for the generalizability of the data. We acknowledge this as a limitation, although we also refer to Eisenhardt [35] in arguing in favor of qualitative case studies, especially in the case of novel research areas. AI ethics, as far as empirical data goes, is a novel area of research. Moreover, the multiple case study approach adds some further validity to the data, as we do not base our arguments on a single case. Nonetheless, another limitation in the data is also that all the cases were based on Finland. For example, the implementation of AI ethics can be more of a focus in US-based companies, as much of the current discussion on AI ethics also originates from the US.

One other limitation in the data is that the interviews were conducted in Finnish. The constructs such as transparency may not carry the same connotations in Finnish as they do in English. This is especially the case with accountability and responsibility, which may not translate in a straightforward manner. However, during the interviews, we sought to clear any misunderstandings related to the constructs with the respondents.

The research framework can also be argued to be a limitation. As AI ethics is a currently active field in terms of theoretical discussion, the constructs in the area are constantly evolving. The ART principles and EAD chosen as a basis of the framework were, at the time of writing, some of the most prominent works in the area. The framework ultimately presents but one way of perceiving AI ethics.

## Conclusions and Future Work

This paper furthers our understanding of the current state of practice in the field of AI ethics. By means of a multiple case study, we studied the way AI ethics is currently implemented in practice, if it is implemented at all, when it is not formally or systematically implemented in software engineering projects.

Our findings can be summarized through the following two key takeaways:

- Even when ethics are not particularly considered, some currently commonly used software development practices, such as documentation, support EAD. This is also the case with focusing on information security.
- While the developers speculate potential socioethical impacts of the resulting system, they do not have means to address them.

Thus, from the point of view of software engineering methods and practices, this highlights a gap in the area. While some of the existing common practices support the implementation of some aspects of AI ethics, there are no methods or practices that help implement it on a project-level.

Further studies on the topic should seek to assist in the practical implementation of AI ethics. Singular practices and especially project-level methods are needed to bridge the gap between research and practice in the area. This lack of higher-level methods was also highlighted in a review of tools and methods in the area [6].

## Footnotes

1 https://yle.fi/uutiset/3-11158701.

2 https://www.reuters.com/article/us-amazon-com-jobs-automation-insight/amazon-scraps-secret-ai-recruiting-tool-that-showed-bias-against-women-idUSKCN1MK08G.

3 ACM Code of Ethics and Professional Conduct. https://www.acm.org/code-of-ethics.

4 http://users.jyu.fi/~vimavakk/AIDevQuestionnaire.
